# Sociodemographic characteristics and vaping motives as potential correlates of early vaping initiation

**DOI:** 10.3389/fpubh.2024.1484252

**Published:** 2025-01-07

**Authors:** Aisha Al-Naimi, Fatma Al-Obaidli, Reem Al-Rashdi, Fatima Al Zahraa Chokor, Mohammed Al-Hamdani

**Affiliations:** Department of Public Health, College of Health Sciences, QU Health, Qatar University, Doha, Qatar

**Keywords:** vaping, sociodemographic variables, early vaping initiation, vaping motives, Middle East

## Abstract

**Background:**

Vaping’s popularity has particularly increased among young people, with its prevalence varying across different regions, including the Middle East. The health impacts of vaping, especially when initiated early, are a growing concern.

**Aims:**

This study aimed to investigate the correlates of early vaping initiation (EVI) and explored the sociodemographic characteristics and vaping motives influencing EVI among vapers from Arab countries.

**Methods:**

An online cross-sectional survey recruited 428regular vapers, aged 18–60 who resided in Arab countries at the time of the study. Sociodemographic and vaping motives data were collected. Stepwise logistic regression was used to examine the factors associated with EVI.

**Results:**

The study findings revealed that older participants and expats have lower odds of EVI. Males and vapers from Qatar had around 4–5 times the odds of EVI as compared to females and those from Egypt, respectively.

**Conclusion:**

Targeted social marketing and education campaigns may benefit groups at risk of EVI, including residents of Qatar, males, and those who are strongly influenced by social media or who have friends or family members who vape. Reducing EVI is particularly important, as vaping often begins at an early age, and early intervention is vital to prevent early initiation and subsequent addiction.

## Background

Vaping involves the inhalation and exhalation of vapor generated by an electronic device which heats a flavored fluid, typically enhanced with nicotine, producing a flavored vapor (1). These devices are compact and rechargeable, making them convenient and appealing to the younger generations. Unlike traditional cigarettes, vaping does not produce a strong odour but emits pleasant fruity or sweet aromas (2). Vaping is predominantly perceived as an alternative to smoking cigarettes, perceiving it as a ‘safer option’ than tobacco (3), despite warnings of its potential for tobacco renormalization and potential harm (4).

The popularity of vaping surged over the years, particularly among younger adolescents globally (5). According to a study, the prevalence of vaping varies across regions. In Europe, prevalence was 14%, one of the highest rates studied. In America, the prevalence was lower at 10% (6). Asia has an 11% prevalence, and Oceania has 6%. In Egypt, a cross-sectional study showed a prevalence of 10.6% among university students (7), while 27.7% of the students in KSA were regular vapers (8). Another survey conducted in six universities in Palestine showed vaping prevalence of 19.7% (9), while the prevalence of vaping was observed to be 14% among Qatar University students (10).

Motives for vaping among adolescents include peer pressure, curiosity, and social approval (11). Personality traits like spontaneity, thrill-seeking, and anxiety sensitivity also influence decisions to vape (12). Another factor is the availability of different flavors (13). Many adolescents vape to experiment, replace cigarettes, or for entertainment (14). In the US, most vapers have a history of smoking (15), used as a non-toxic alternative to quit smoking (16). Social media platforms, like Twitter, TikTok, and Instagram have influenced vaping habits (17). Moreover, lower education levels correlate with less awareness of vaping harms and, therefore, higher use of vape (18).

Vaping initiation increases the likelihood of cigarette use, leading to nicotine addiction and cancer (19). Early nicotine exposure can impair brain development and affect bone development, lungs, and ocular health (20). Adolescent vapers are more prone to respiratory symptoms, as well as cardiovascular, developmental, and immunologic issues (21, 22). It also poses a risk to children exposed to vaping environments, increasing their chances of experiencing toxic effects like such as nausea, convulsions and respiratory symtoms (23).

### Current gaps and study objective

Given the widespread use of vaping and its health risks, it is crucial to examine the factors that are associated with early initiation of vaping. Although previous research has focused adolescents as the target population for EVI, this study takes a retrospective approach, focusing on adults aged 18 and older. By including regular vapers from this age group, we aim to capture individuals who initiated vaping before the age of 18, thus allowing us to explore the factors contributing to EVI. As far as we know, no research has investigated the correlates of EVI in the Arab region. This study, therefore, aims to explore the correlates of early vaping initiation among regular vapers aged 18 and older in a sample of Arab countries.

## Methods

### Data collection

This study is based on data collected between February and May 2023 by a cross-sectional online survey using the Blue online survey platform. A link to the survey was posted and boosted on social media platforms to reach users.

### Study sample

Eligibility criteria for the survey included being a social media user aged 18–60, being a regular vaper who uses any vaping device at least once a week for no less than 3 months and residing in an Arab country. Exclusion criteria were age, under 18 or over 60, not being a regular vaper, or not residing in an Arab country. Participants who completely answered the questions of interest were included in the study.

### Ethical approval

The survey received ethical clearance from Qatar University [#QU-IRB 1806-E/23]. An electronic informed consent form, highlighting the study’s purpose, potential harms, benefits, confidentiality measures, and data storage procedures was presented in the online survey. Only individuals who agreed to participate after reviewing the consent form were able to take part in the study.

### Survey measures

The survey included questions about the respondent’s sociodemographic characteristics such as age, country of residence (Qatar, Iraq, Egypt, Other), gender, and residence status (citizen, expat). It also included questions about vaping behaviors such as age at which the respondent started vaping (in years), strongest influence to start vaping (wanting to quit smoking versus friends, family, or social media), using flavored juice when started vaping (yes, no), type of vape juice used when started vaping (with or without nicotine), and smoking before vaping (yes, no) (12).

The Modified Drinking Motives Questionnaire-Revised Short Form scale was used to assess the coping, sensory, cognitive, enhancement, and social motives for starting vaping. The questionnaire, adopted from Davidson et al. (12) and modified from the original Woicik et al. scale (24), presented statements/items related to vaping motives for which participants chose from the scale: 1 “always/almost always,” 2 “most of the time,” 3 “half of the time/some of the time,” and 4 “never/almost never,” to indicate how frequently their vaping is motivated by each of the reasons listed. For the analysis of this study, only subscales that showed high reliability were included (enhancement motive and social motive subscales). The survey was pilot-tested on five participants to ensure simplicity and clarity.

### Sample size calculation

G*power was conducted to identify the required sample size. A minimum sample size of 308 was needed to detect an odds ratio (OR) ≥1.5 at an alpha level of 0.05 and power = 0.8 for a two-tailed logistic regression analysis.

### Statistical analysis

The reported age at which the respondent started vaping was categorized into two groups: early initiation of vaping if the age was 18 years or below versus not early initiation of vaping if the age was above 18 years. Descriptive statistics for the respondent’s sociodemographic characteristics and vaping motives were presented as medians and interquartile ranges (IQR) for non-normally distributed continuous variables, as well as frequencies and percentages for categorical variables. The normality of continuous variables was assessed through the use of histograms and Q-Q plots. Categorical and continuous variables were compared between participants who initiated vaping at an early age versus those who did not use Chi-square tests and the non-parametric test Mann–Whitney U tests, respectively. Binary logistic regression was used to test the correlates of early initiation of vaping (the dependent binary variable defined as yes versus no). The relationships of sociodemographic characteristics, vaping-related characteristics, and vaping motives were further explored using backward stepwise variable selection for a multiple binary logistic regression analysis to identify significant independent correlates of early initiation of vaping. A *p*-value cut-off of 0.1 and 0.2 was set for model entry and removal, respectively. Odds ratio (OR) was used to report the findings along with the 95% confidence interval (CI). To check for multicollinearity among independent variables, we used the variance inflation factor (VIF) cut-off of VIF > 10 as the threshold for collinearity. Data analysis was carried out using StataSE 18. Statistical significance was assessed at an alpha level of 0.05.

## Findings

### Summary of sample characteristics

Sociodemographic and vaping characteristics are displayed in Table 1 for the total sample by EVI status. The sample consisted of 428 regular vapers aged between 18 and 60 years. The median age for the participants was 26 years; 8.6% were females, and 91.4% were males. Additionally, less than half of the participants (44.9%) were from Egypt, 33.4% from Iraq, 11.9% were from Qatar, and 9.8% were from other countries, including Lebanon, Syria, Jordan, Palestine, Oman, Sudan, Yemen, Kuwait, Bahrain, Saudi Arabia, and the United Arab Emirates. Most participants were citizens of their respective countries (89.5%), while 10.5% were expatriates. More than half of the participants (57.5%) reported that “wanting to quit smoking” was their primary reason for vaping initiation. In comparison, 42.5% reported family, friends, and social media as the strongest influence to start vaping. Moreover, 92.8% of the participants used a flavored vaping product when they started vaping, and 14.5% of the participants began to use vaping products without nicotine. Furthermore, 78.5% started smoking before vaping.

**Table 1:** Sociodemographic and vaping characteristics of regular vapers by early vaping initiation status.

	No early initiation of vaping (*n* = 331) *N* (%)	Early initiation of vaping (*n* = 97) *N* (%)	Total sample (*n* = 428) *N* (%)	*p*-value^*^
Age (years)ǂ	30 (13)	21 (4)	27 (13.5)	<0.001+
Sex				
Female	23 (62.2)	14 (37.8)	37 (8.6)	0.021+
Male	308 (78.8)	83 (21.2)	391 (91.4)	
Country				
Egypt	162 (84.4)	30 (15.6)	192 (44.9)	<0.001+
Iraq	114 (79.7)	29 (20.3)	143 (33.4)	
Qatar	28 (54.9)	23 (45.1)	51 (11.9)	
Others^1^	27 (64.3)	15 (35.7)	42 (9.8)	
Residence				
Citizen	297 (77.6)	86 (22.5)	383 (89.5)	0.763
Expat	34 (75.6)	11 (24.4)	45 (10.5)	
Age of vaping initiation (years) ǂ	26 (12)	17 (2)	23 (11)	<0.001+
Strongest influence to start vaping			
Wanting to quit smoking	212 (86.2)	34 (13.8)	246 (57.5)	<0.001+
Friends, family, or social media	119 (65.4)	63 (34.6)	182 (42.5)	
Used a flavored vape juice at initiation				
No	24 (77.4)	7 (22.6)	31 (7.2)	0.991
Yes	307 (77.3)	90 (22.7)	397 (92.8)	
Type of vape juice at initiation			
With nicotine	290 (79.2)	76 (20.8)	366 (85.5)	0.023+
Without nicotine	41 (66.1)	21 (33.9)	62 (14.5)	
Smoking before vaping				
No	56 (60.9)	36 (39.1)	92 (21.5)	<0.001+
Yes	275 (81.9)	61 (18.1)	336 (78.5)	
Enhancement motive scale ǂ	2 (2)	2.3 (2.7)	2 (2.3)	0.0651
Social motive scale ǂ	3.7 (2)	3.7 (1.7)	3.7 (1.7)	0.3713

*p-values were obtained from chi-square tests for categorical variables, as applicable or using Mann–Whitney U tests for continuous variables.

^ǂ^Continuous variables (age, age of initiation, enhancement motive scale, and social motive scale) are summarized using medians and interquartile ranges.

^1Others include Lebanon, Syria, Jordan, Palestine, Sudan, Yemen, KSA, Kuwait, UAE, Oman, and Bahrain.^

^+^Significant values.

### Main results

Table 1 also shows that differences were found between those who started vaping at an early age versus those who did not in terms of sex, age, and country of residence. Age distribution was statistically lower among those who initiated vaping at an early age as compared to those who did not (*p* < 0.001). Females had a significantly higher proportion of EVI (37.8%) than males (21.2%). Moreover, participants residing in Qatar had a higher proportion of EVI (45.1%) as compared to those living in Egypt (15.6%) (*p* < 0.001). The median age of vaping initiation across the entire sample was 23 years, with an IQR of 11. Notably, early vaping initiators exhibited significant differences in their age of initiation (median = 17 years) compared to non-early initiators (median = 26 years) (*p* < 0.001). Moreover, as compared to those who started vaping because they wanted to quit smoking, those whose strongest influence to start vaping were friends, family, or social media had significantly higher proportions of EVI (*p* < 0.001).

Further, participants who started vaping using vape juice with nicotine had a significantly lower proportion of EVI (20.8%) compared to those who started using vape juice without nicotine (33.9%) (*p* = 0.023). Similarly, participants who smoked before vaping had a lower prevalence of EVI (18.1%) compared to those who did not smoke before vaping (39.1%) (*p* < 0.001).

Table 2 illustrates the results of logistic regression analyses with the EVI (yes/no) as the dependent variable. One year increase in age is significantly associated with lower odds of EVI by 30% (OR: 0.7, 95% CI: 0.6, 0.8). Males and those residing in Qatar had four times the odds of EVI compared to females and those in Egypt, respectively. Moreover, expats had lower odds of EVI than citizens by 80% (OR = 0.2, 95% CI: 0.1, 0.7). It is worth mentioning that those who reported having friends, family, or social media as the strongest influence to start vaping had approximately two times the odds of EVI as compared to those who started vaping because they wanted to quit smoking (borderline significance). Vaping characteristics and motive scales were not retained in the final model based on the backward stepwise selection.

**Table 2:** Simple and multiple logistic regressions for the correlates of early initiation of vaping among 18–60 years old regular vapers.

	Crude OR (95% CI)	Adjusted OR (95% CI)^*^
Age (years)	0.7 (0.7, 0.8)	0.7 (0.6, 0.8)
Sex		
Female	Reference	Reference
Male	0.4 (0.2, 0.9)	4.2 (1.4, 12.7)
Country		
Egypt	Reference	Reference
Iraq	1.4 (0.8, 2.4)	0.6 (0.3, 1.3)
Qatar	4.4 (2.2, 8.7)	4.5 (1.2, 16.3)
Others^1^	3.0 (1.4, 6.3)	1.3 (0.5, 3.6)
Residence		
Citizen	Reference	Reference
Expat	1.1 (0.5, 2.3)	0.2 (0.1, 0.7)
Strongest influence to start vaping		
Wanting to quit smoking	Reference	Reference
Friends, family, or social media	3.3 (2.1, 5.3)	1.8 (1.0, 3.3)
Used a flavored vape juice at initiation		
No	Reference	
Yes	1.0 (0.4, 2.4)	
Type of vape juice at initiation		
With nicotine	Reference	
Without nicotine	2.0 (1.1, 3.5)	
Smoking before vaping		
No	Reference	
Yes	0.3 (0.2, 0.6)	
Enhancement motive scale	1.2 (1.0, 1.5)	
Social motive scale	1.1 (0.9, 1.4)	

Significant ORs are displayed in red font.

^*^Based on backward stepwise selection regression model using likelihood ratio test.

^1Others include Lebanon, Syria, Jordan, Palestine, Sudan, Yemen, KSA, Kuwait, UAE, Oman, and Bahrain.^

Sensitivity analyses were applied to the 20–34 years old subsample since they are most impacted by vaping initiation. Another set of analyses were applied to vapers who are citizens only to check whether the convergence of country and residence status impacts vaping initiation. The results of both analyses yielded similar effects to the overall sample concluding that neither a focus on a narrower age group nor the convergence of country and residence status are differentially related to vaping initiation (see Supplemental File).

## Discussion

Our study found that age, sex, country of residence, residence status, and influences from family, friends, and social media (borderline significance) are significantly associated with EVI. These findings align with existing literature, where several studies have reported a higher prevalence of vaping among young adolescents than older ones (25, 26). The lack of understanding and knowledge of the potential health implications of vaping might explain this higher prevalence among the younger ones (27). Moreover, the negative association between age and EVI suggests that younger vapers are more likely to have started vaping earlier compared to older vapers. This could reflect the increasing prevalence of vaping among the younger generation, as vaping has gained popularity in more recent years, coinciding with increased availability and marketing of e-cigarettes. In addition, sex differences in EVI were also observed, with males tending to begin vaping at a younger age than females. This trend is consistent with previous studies showing historically higher rates of tobacco use among males of all ages (28). Additionally, studies have shown that males are frequently the first to adopt new technologies, and vape products are no exception (29). The increased likelihood of males using vape may be attributed to their lower perception of harm associated with vaping (3, 16).

Further, the country of residence is also associated with EVI, with residents of Qatar showing higher odds of EVI. A previous study reported that 14% of Qatar College undergraduates vape (10). Individuals in higher-income countries are more prone to spending their income on vaping devices and, therefore, have more access to resources (30). Generally, citizens have higher incomes than expatriates, which may explain why citizens are more likely to initiate vaping early (31). Likewise, friends, family, and social media have a borderline significant association with EVI. This finding is consistent with literature indicating that having a family member who vapes increases the likelihood of vaping among adolescents (32). Adults with friends who view vaping positively are more likely to vape (25). Additionally, promotion of vaping on social media has a significant influence on young adolescents (33).

### Implications

The results of the current study have several important implications. First, caution should be exercised to prevent EVI, particularly with male adolescents. This can be addressed through parental support or by engaging males in extracurricular activities to develop better coping mechanisms. Engaging them in skill acquisition to refuse vaping products when offered can be crucial in early intervention (34).

Secondly, the higher likelihood of EVI for those residing in Qatar suggests that being cautious about youth spending is necessary to prevent EVI. Parents should consider reducing allowances or buying items for young adolescents rather than giving them money to avert EVI. Additionally, parents can provide gift cards from places that do not sell vaping products, as lower affordability is associated with reduced vaping, particularly among youth (11).

Our study’s findings also highlight the need for regulatory measures to restrict the exposure of young social media users to vaping content. This is crucial as past research has shown that social media influences vaping behavior and increases the likelihood of vaping. Therefore, it is prudent to limit the exposure to vaping content for individuals under the age of 18 (19).

### Strengths and limitations

To our knowledge, this research addresses an understudied area of vaping literature, which is the correlates of EVI. Our study adds to the scarce literature on vaping control in the Middle East. However, our research has some limitations. The cross-sectional design does not test causal relationships between sociodemographic and vaping motive correlates and EVI. A longitudinal study may be necessary to explore the causal relationships between various correlates and EVI. Additionally, this study included social media users only from a limited number of Arab countries, which limits the generalizability to all regular vapers in the Arab countries. Social media users, especially those who engage in online surveys, may differ from those who do not use these platforms in terms of sociodemographic characteristics (e.g., age, education, socioeconomic status), potentially leading to selection bias. Furthermore, there is a possibility some participants would respond by either underestimating or overestimating their responses, or inaccurately recalling or misreporting their vaping behaviors introducing social-desirability bias. Moreover, because the sample consisted of participants aged 18 and older, the retrospective reporting of vaping initiation before the age of 18 might be subject to recall bias, especially in older respondents. Additionally, the majority of the studied vapers in this study were not early vaping initiators. This limitation can be rectified in future studies via recruiting a larger portion of early vaping initiators in studied samples. It is also important to note that this study grouped vaping initiators into two groups: EVI (<18 years old) and non-EVI (≥18 years). This way of grouping vapers does not distinguish between EVI at different stages of adolescence (early vs. late adolescence) which does not allow for correlating sociodemographic variables with EVI at different stages of adolescence. Future studies can divide EVI by stage of adolescence and compare how each stage differs from non-EVI with respect to sociodemographic correlates to offer more nuanced interpretation. Finally, we examined a limited number of predictors, while other factors such as parent’s education level and the presence of mental health disorders may also affect EVI.

## Conclusion

This study suggests that sociodemographic characteristics such as sex, country of residence, residence status, and social influences from friends, family, or social media are significantly associated with EVI. Notably, younger age groups had higher odds of EVI, which may highlight emerging trends within younger populations. Future research and policy-making efforts shall, therefore, aim at mitigating the rise of vaping, particularly among younger adults. Further studies are encouraged to explore interventions and preventive measures that address these early initiation trends. Future studies could also expand on these findings by investigating other risk factors and longitudinal patterns, helping to deepen our understanding of vaping behavior in the broader population.

## Data Availability

The datasets presented in this article are not readily available due to the sensitive nature of the data. The anonymized data will only be retained for 5 years from the end date of data collection as this was a condition stated in the informed consent. Requests to access the datasets should be directed to malhamdani@qu.edu.qa.
