# Supplementary material for: Sociodemographic characteristics and vaping motives as potential correlates of early vaping initiation
Source: Front Public Health. 2025 Jan 7;12:1484252. doi: 10.3389/fpubh.2024.1484252 (PMC11747700; doi:10.3389/fpubh.2024.1484252)
Supplement: Supplementary file 1 [file Table_1.docx]

**APPENDIX 1**

Multiple logistic regression for the correlates of early initiation of vaping among 20-34 years old regular vapers *(n=265) and among citizens (n=383)*

|  | **Among 20-34 years old regular vapers *(n=265)*** | ***Among citizens (n=383)*** |
| --- | --- | --- |
|  | ***Adjusted OR (95% CI)**** | |
| *Age (years)* | 0.7 (0.6, 0.8) | 0.7 (0.6, 0.8) |
| Sex |  |  |
| *Female* | Reference | Reference |
| *Male* | 8.3 (1.9, 35.2) | 6.5 (1.5, 28.0) |
| Country |  |  |
| *Egypt* | Reference | Reference |
| *Iraq* | 0.8 (0.3, 1.8) | 0.6 (0.3, 1.3) |
| *Qatar* | 7.7 (1.7, 34.9) | 7.8 (1.6, 38.2) |
| *Others¹* | 1.7 (0.5, 5.8) | 1.6 (0.5, 4.5) |
| Residence |  |  |
| *Citizen* | Reference |  |
| *Expat* | 0.2 (0.04, 0.7) |  |
| Strongest influence to start vaping |  |  |
| *Wanting to quit smoking* |  | Reference |
| *Friends, family, or social media* |  | 1.7 (0.9, 3.2) |

*Notes. Significant ORs are displayed in red font.*

**Based on backward stepwise selection regression model using likelihood ratio test*

*¹ Others include Lebanon, Syria, Jordan, Palestine, Sudan, Yemen, KSA, Kuwait, UAE, Oman, and Bahrain.*
